# Epidemiology and clinical outcomes of hospitalized Hispanic patients with IBD: results of a large national cohort study

**DOI:** 10.1007/s00384-025-04822-z

**Published:** 2025-02-14

**Authors:** Alex Zhornitskiy, Felicia Zhornitsky, Waqas Rasheed, Eric J. Mao

**Affiliations:** 1 Department of Gastroenterology and Hepatology, University of California Davis Medical Center, 4150 V St, Suite 3500, Sacramento, CA 95817 USA; 2 School of Medicine, University of California, Davis, Sacramento, CA USA; 3 Department of Medicine, University of Kentucky, Lexington, KY USA

**Keywords:** Hispanic, Ethnicity, Inflammatory bowel disease, Ulcerative colitis, Crohn’s disease

## Abstract

**Introduction:**

Inflammatory bowel disease (IBD) has historically been seen as predominantly affecting non-Hispanic Whites (NHW). Hispanics are the largest minority group in the USA, yet they remain grossly underrepresented in studies of IBD. With this study, we aimed to better understand the epidemiology of hospitalized Hispanic patients with IBD in the US.

**Methods:**

This was a retrospective cohort study utilizing the National Inpatient Sample, the largest publicly available all-payer inpatient care database in the United States. We compared demographics, hospitalization characteristics, clinical outcomes, and year-to-year trends from 2016 to 2020 in Hispanic and NHW with a primary diagnosis of inflammatory bowel disease, Crohn’s disease, or ulcerative colitis.

**Results:**

NHWs hospitalized with a primary diagnosis of IBD had significantly higher rates of hospitalization than Hispanics (122.67 vs 71.12, *P* < 0.01). While hospitalized Hispanics with IBD are more likely to be in the lowest quartile for household income (31.6% vs 19.3%, *P* < 0.01), have a younger median age (37.0 vs 45.0, *P* < 0.01), and be uninsured (4.3% vs 8.8%, *P* < 0.01) compared to NHW. Length of admission was similar, yet NHWs had higher rates of mortality (0.3% vs 0.2%, *P* = 0.01), while total charges for hospitalizations were significantly higher for Hispanic patients (*P* < 0.01).

**Discussion:**

To our knowledge, this is one of the largest US-based studies of Hispanics with IBD. Our findings suggest that among hospitalized IBD patients, Hispanics are more likely to be younger, uninsured, have a lower household income, and are less likely to undergo surgery while having higher hospital charges.

## Introduction

Historically inflammatory bowel disease (IBD) has been viewed as a condition that afflicts predominantly Western Europeans. However, in the past several decades, there have been a multitude of peer-reviewed manuscripts that challenge this notion as they show that populations at traditionally lower risk were experiencing an increasing incidence of IBD globally [1]. There have been similar attempts to better characterize the epidemiology of IBD in the United States (US), but these studies disproportionately evaluated non-Hispanic White (NHW) populations, and thus, their advancement of our understanding of IBD in minority populations is limited [2–4]. This lack of ethnoracial heterogeneity impairs our understanding of the epidemiology, demographics, and medical/surgical treatment outcomes for these diseases among minorities.

Some smaller, single-institution studies have shown not only different clinical phenotypes but also interestingly enough that the incidence of at least UC is no different among Hispanics compared to NHWs [5, 6]. Such studies are not only re-shaping but also challenging the core tenets of our epidemiological understanding of IBD. However, despite being not only the largest but also the fastest-growing minority group in the US, there remains little national data showing adequate representation of Hispanics in IBD research [7].

In our study, we looked to better understand the epidemiology, medical and surgical inpatient management, and overall hospitalization course of Hispanics relative to NHWs.

## Methods

### Design and data source

Design and data source: This retrospective cohort study used data from the National Inpatient Sample (NIS) database, which is the largest publicly available all-payer inpatient care database in the United States. The NIS contains data from approximately 7 million hospital stays each year, representing a 20% stratified sample of US community hospitals. We obtained data from the NIS for the years 2016–2020 using the International Classification of Diseases, Tenth Revision, and Clinical Modification (ICD-10-CM) codes to identify hospitalizations with the primary diagnosis of IBD (I10_DX1) (Crohn’s disease or ulcerative colitis). Patient characteristics, including demographics, co-morbidities, and hospital-related factors, were extracted from the database. Additional information on the design and sampling methods of the NIS is available at https://www.hcup-us.ahrq.gov.

### Study population and outcome measures

We compared hospitalization characteristics among the three racial groups, including patient demographics, co-morbidities, length of stay (LOS), and hospital costs. Additionally, we looked at clinical outcomes like in-hospital mortality, complications, and the requirement for surgical interventions. We examined trends in hospitalization rates, costs, and length of stay (LOS) to determine the impact of IBD on the US healthcare system.

### Statistical analysis

The statistical analysis was conducted using version 16.0 of the STATA software (StataCorp LLC, Station, TX, USA) to examine the patient characteristics. The results were presented as frequency (*N*) and percentage (%) for categorical variables and medians with interquartile range (IQR) for continuous variables, as appropriate. The number of hospitalizations per year was weighted to provide a nationwide estimate in accordance with the recommendations of the AHRQ. We analyzed the differences in continuous variables across groups using non-parametric statistical tests due to the non-normal distribution of the data. The Chi-squared test was employed for categorical variables to compare the characteristics of the patients as well as medians for length of stay. Additionally, we used the Cuzick test, a non-parametric test for trends across ordered groups implemented in Stata through the nptrend command, to assess the presence of a trend in the median of continuous variables across the years. It is important to note that statistical significance may only sometimes imply practical significance. To investigate the relationship between the dependent variables and race over time, we used linear regression analysis. An interaction term between race and year was included to compare the trend of the variables over time among the two racial groups (compare slopes of two racial groups across time) and to determine whether there were any significant differences in the rate of change in the dependent variables over time. The level of significance for all tests was set at 0.05, and *P*-values were two-sided.

## Results

### Demographics

The total number of hospitalizations of NHW patients with IBD over the course of 5 years was significantly greater (*P* < 0.01) than Hispanics (Table 1). However, more pertinent are the rates of IBD hospitalizations per 100,000 from each respective ethnic group given the intrinsic difference in number of NHW and Hispanics in the database (69.8% and 13.6%, respectively). The rates of hospitalizations per 100,000 for a primary admission diagnosis of IBD, UC, and CD (*P* < 0.01) were significantly higher in NHW relative to Hispanics. Given the ethnicities that make up the term Hispanic varying significantly depending on the region of the United States that is being evaluated, we stratified the Hispanic population we were evaluating by region (Table 2). Interestingly, this showed significantly higher rates of IBD, CD, and UC hospitalizations among Hispanics in the Northeast (*P* < 0.01).

**Table 1:** Demographics of inflammatory bowel disease hospitalizations for NIS database 2016–2020

	**Inflammatory bowel disease**			**Crohn’s disease**			**Ulcerative colitis**		
	**White**	**Hispanics**	***P*-value**	**White**	**Hispanics**	***P*-value**	**White**	**Hispanics**	***P*-value**
Total hospitalizations	133,305	15,055	< 0.01	87,330	7225	< 0.01	45,975	7830	< 0.01
Rate per 100,000 NIS hospitalizations	122.67	71.12	< 0.01	80.36	34.13	< 0.01	42.31	36.99	0.03
Gender (%Female)	70,640 (53.0%)	7660 (50.9%)	< 0.01	45,595 (52.2%)	3565 (49.3%)	< 0.01	25,045 (54.5%)	4095 (52.3%)	0.02
Age in years, median	45.0	37.0	< 0.01	44.0	37.0	< 0.01	48.0	37.0	< 0.01
Age group (years)									
0–17	7315 (5.5%)	1575 (10.5%)	< 0.01	3925 (4.5%)	680 (9.4%)	< 0.01	3390 (7.4%)	895 (11.4%)	< 0.01
18–64	97,810 (73.4%)	11,155 (74.1%)	0.06	67,045 (76.8%)	5570 (77.1%)	0.53	30,765 (66.9%)	5585 (71.3%)	< 0.01
≥ 65	28,180 (21.1%)	2325 (15.4%)	< 0.01	16,360 (18.7%)	975 (13.5%)	< 0.01	11,820 (25.7%)	1350 (17.2%)	< 0.01
Median household income									
1st quartile (lowest)	25,355 (19.3%)	4665 (31.6%)	< 0.01	16,690 (19.4%)	2135 (30.0%)	< 0.01	8665 (19.1%)	2530 (32.9%)	< 0.01
2nd quartile	34,245 (26.0%)	3770 (25.5%)	0.08	22,295 (25.9%)	1870 (26.3%)	0.52	11,950 (26.4%)	1900 (24.7%)	0.01
3rd quartile	36,110 (27.5%)	3740 (25.3%)	< 0.01	23,915 (27.7%)	1790 (25.2%)	< 0.01	12,195 (26.9%)	1950 (25.4%)	0.02
4th quartile (highest)	35,800 (27.2%)	2610 (17.7%)	< 0.01	23,310 (27.0%)	1310 (18.4%)	< 0.01	12,490 (27.6%)	1300 (16.9%)	< 0.01
Insurance type (%)									
Medicare	35,275 (27.3%)	2690 (18.7%)	< 0.01	22,015 (26.0%)	1220 (17.6%)	< 0.01	13,260 (29.8%)	1470 (19.7%)	< 0.01
Medicaid	18,390 (14.2%)	4700 (32.7%)	< 0.01	12,400 (14.7%)	2050 (29.6%)	< 0.01	5990 (13.5%)	2650 (35.5%)	< 0.01
Private	69,820 (54.1%)	5715 (39.8%)	< 0.01	46,710 (55.2%)	3075 (44.4%)	< 0.01	23,110 (52.0%)	2640 (35.4%)	< 0.01
Uninsured	5590 (4.3%)	1270 (8.8%)	< 0.01	3490 (4.1%)	575 (8.3%)	< 0.01	2100 (4.7%)	695 (9.3%)	< 0.01

*NIS* National Inpatient Sample, *NHW* non-Hispanic White

**Table 2:** Rates per 100,000 NIS hospitalizations of Hispanic patients w/ IBD depending on region

	**Northeast**	**Midwest**	**South**	**West**	***P*-value**
IBD	108.94	71.76	73.43	53.30	< 0.01
Crohn’s disease	61.82	34.79	36.26	20.62	< 0.01
Ulcerative colitis	47.12	36.96	36.96	32.68	< 0.01

*NIS* National Inpatient Sample, *IBD* inflammatory bowel disease

Also notably, there is a significant gender difference, with Hispanics hospitalized with IBD being more likely to be women (53.0% vs 50.9%, *P* < 0.01) (Table 1). A significant age difference was also appreciated between the two groups with hospitalized Hispanic patients more likely to have a younger median age, and more likely to be < 18 years old (*P* < 0.01). Meanwhile, NHW patients are more likely to be > 65 years old (*P* < 0.01). Median household income was evaluated by quartiles with Hispanics being significantly more likely to be in the lowest income quartile (*P* < 0.01), NHWs being significantly more likely to be in the highest income quartile (*P* < 0.01), and no significant difference between the two in the middle two quartiles. Lastly, it was notable that in terms of types of insurance, Hispanics were more likely to have Medicaid or be uninsured (*P* < 0.01), while NHWs were more likely to have Medicare or private insurance (*P* < 0.01).

### Associated diagnoses and hospitalization characteristics

While there was no difference in rates of *Clostridium difficile* (*C. diff*) infections in hospitalizations for IBD or CD, NHWs admitted for UC had higher rates of *C. diff* infection than Hispanics (Table 3). No data on biologic use was able to be obtained; however, steroid use was also noted to be significantly higher in NHWs (*P* < 0.01). In terms of associated medical conditions, primary sclerosing cholangitis (PSC) was significantly more likely to be seen in NHWs as compared to Hispanics (*P* < 0.01). Surgical intervention whether during or prior to the hospitalization was significantly higher in NHWs relative Hispanics, notably NHWs had higher rates of small bowel surgery among CD hospitalizations (*P* < 0.01) and total colectomies among UC hospitalizations (*P* < 0.01).

**Table 3:** Associated diagnoses and hospitalization characteristics

	**Inflammatory bowel disease**			**Crohn’s disease**			**Ulcerative colitis**		
	**White**	**Hispanics**	***P*-value**	**White**	**Hispanics**	***P*-value**	**White**	**Hispanics**	***P*-value**
Steroid use	10,450 (7.8%)	985 (6.5%)	< 0.01	6480 (7.4%)	430 (6.0%)	< 0.01	3970 (8.6%)	555 (7.1%)	< 0.01
Small bowel surgery	2150 (1.6%)	115 (0.8%)	< 0.01	2110 (2.4%)	115 (1.6%)	< 0.01	40 (0.1%)	0 (0.0%)	0.01
Partial colectomy	6485 (4.9%)	360 (2.4%)	< 0.01	5570 (6.4%)	245 (3.4%)	< 0.01	915 (2.0%)	115 (1.5%)	< 0.01
Total colectomy	1915 (1.4%)	190 (1.3%)	0.33	35 (< 1%)	0 (0.0%)	0.09	1880 (4.1%)	190 (2.4%)	< 0.01
Rectal surgery	3085 (2.3%)	265 (1.8%)	< 0.01	425 (0.5%)	15 (0.2%)	< 0.01	2660 (5.8%)	250 (3.2%)	< 0.01
ICU level admission	370 (0.3%)	40 (0.3%)	0.91	210 (0.2%)	20 (0.3%)	0.55	160 (0.3%)	20 (0.3%)	0.21
Length of stay, median (days)	3.0	3.0	0.86	3.0	3.0	0.94	4.0	4.0	0.84
Died during hospitalization	375 (0.3%)	25 (0.2%)	0.01	UTR	UTR	NA	220 (0.5%)	20 (0.3%)	0.01
Total charges, median (dollars)	30,548.0	37,417.0	< 0.01	28,616.0	34,390.5	< 0.01	34,095.0	40,869.0	< 0.01

*NIS* National Inpatient Sample, *NHW* non-Hispanic White, *UTR* unable to report (as per NIS guidelines when number is greater than 0 but less than 10), *NA* not applicable

Length of stay of hospitalizations was not significantly different regardless of the primary diagnoses evaluated, and similarly, there was no difference in the frequency of intensive care unit (ICU) admissions. While there were < 10 hospitalizations during the time frame evaluated that resulted in mortality among CD hospitalizations and thus per NIS guidelines are not allowed to be reported in data analysis to ensure patient anonymity, NHWs with UC hospitalizations had significantly higher rates of mortality than Hispanics (0.5% vs 0.3%, *P* < 0.01). Lastly, hospitalization charges were significantly higher in Hispanics with a primary diagnosis of IBD or CD (*P* < 0.01) and no significant difference in UC (*P* = 0.059).

### Hospitalization frequency and charges evaluated yearly

**Fig. 1:**
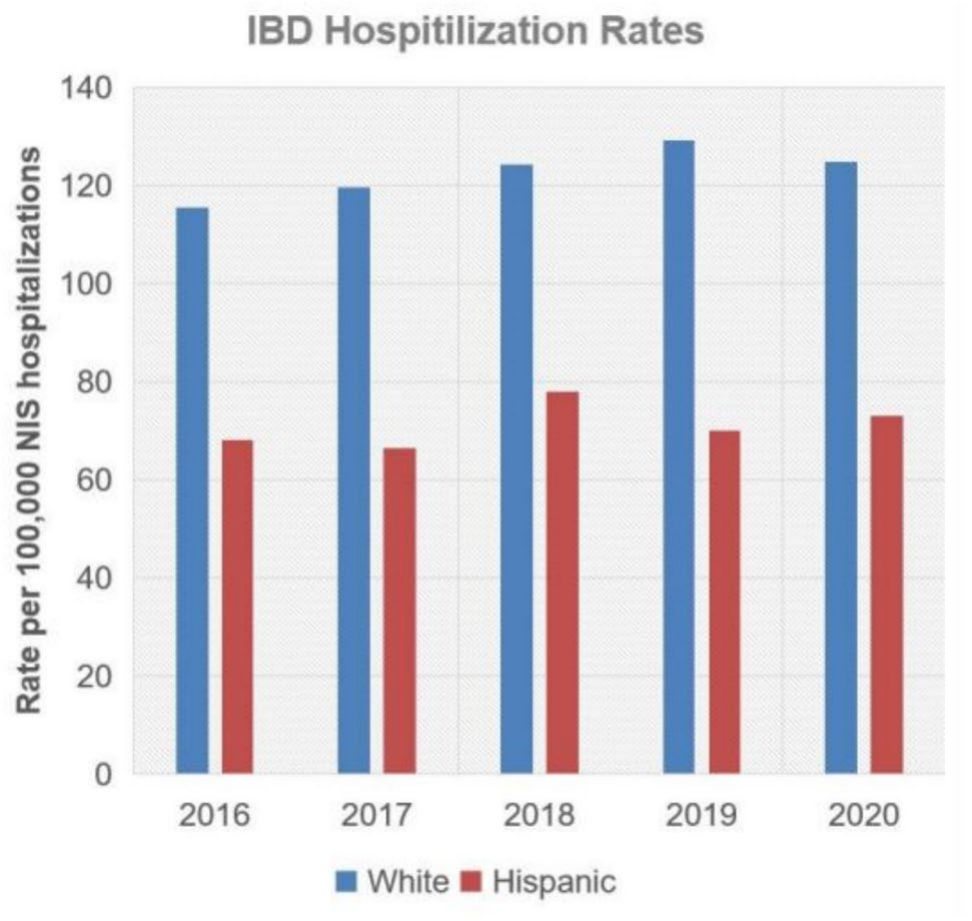
Annual IBD hospitalization rates. IBD, inflammatory bowel disease; NIS, National Inpatient Sample

We further examined on a year-by-year basis with a comparison of the rates of change between the two groups. Notably, rates of hospitalizations among NHWs with IBD grew at a significantly faster rate than among Hispanics (*P* < 0.01) (Fig. 1). A similar significant difference was appreciated among NHWs with UC (*P* < 0.01); however, in those with CD, there appears to have been a significant decline in the rate of hospitalizations among NHWs compared to Hispanics.

**Fig. 2:**
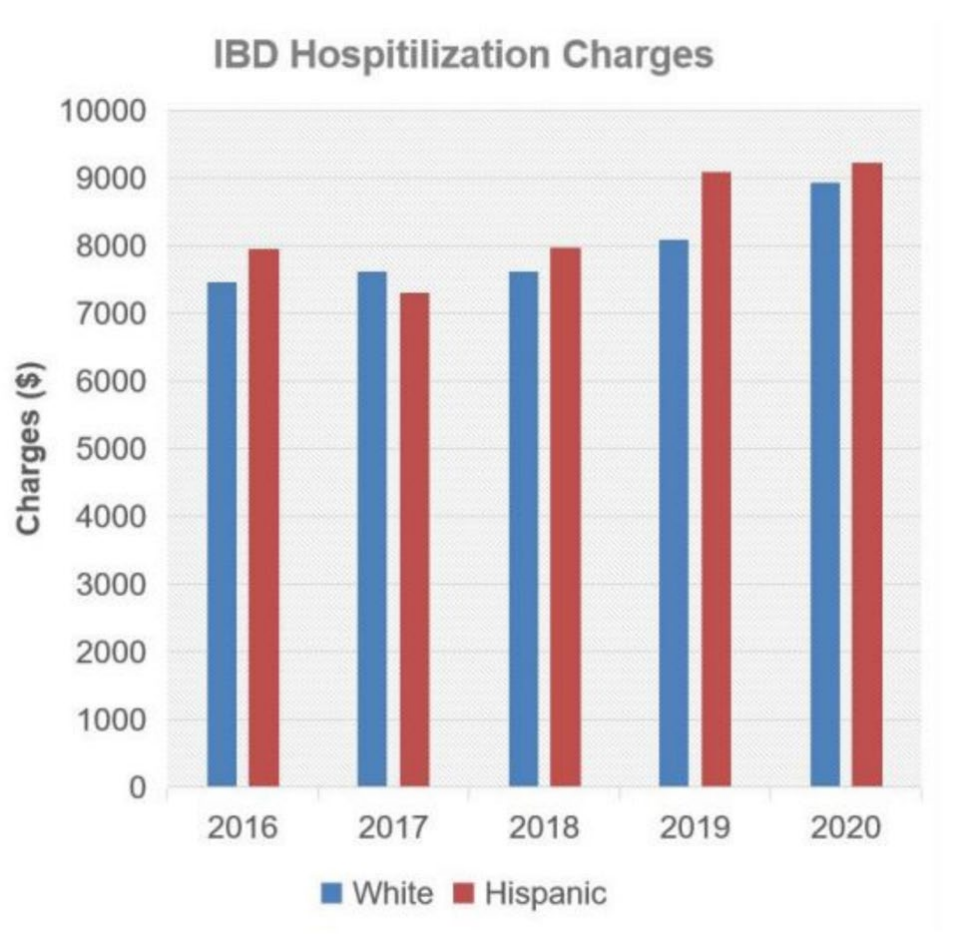
Annual IBD hospitalization charges. IBD, inflammatory bowel disease

Meanwhile, there appears to be a significant increase in hospital charges among NHWs with IBD relative to Hispanics (*P* < 0.01); this difference was not significant in CD (*P* = 0.97) or UC (*P* = 0.66), respectively (Fig. 2).

## Discussion

Despite an extensive amount of research related to IBD in the United States, there remains a paucity of data on IBD among Hispanics as historically US population studies have been composed predominantly of NHWs. Our goal was to better characterize the epidemiology and outcomes of Hispanics hospitalized with IBD. To date, this is one of the largest cohorts of Hispanics with IBD studied in the United States, and our findings help shed light on the differences, as well as some similarities, that exist between Hispanics and NHWs.

While the incidence and prevalence of IBD among Hispanics have been shown to be similar to NHWs in several smaller studies, rates of hospitalizations between the two groups appear to be significantly different in our study [5, 6]. Among patients with IBD, whether it was UC or CD, we found that non-Hispanic Whites had significantly higher rates of hospitalization than Hispanic patients. This finding corresponds to a similar study by Galoosian et al. that predates the time frame we investigated by looking at NIS data from 2007–2013 [8]. However, no prior study to our knowledge has evaluated the rate of change year-to-year in IBD hospitalizations among Hispanics compared to NHWs. While both groups saw an increase in hospitalization rates, among NHWs, it was rising at a significantly greater rate compared to Hispanics. It is unclear what the cause of this may be based on current literature, with postulations including a possible lower threshold for referral to the hospital among NHWs due to their better access to healthcare or increasing frequency of IBD flares refractory to outpatient management, both of which would need further investigation.

Our study also found that among hospitalized patients with IBD, Hispanics are significantly younger and more likely to be women. While the prevalence of IBD has been shown to be higher in adult women, in a recent study by Lewis et al. utilizing multiple large datasets, there has been limited data regarding differences between Hispanics compared to NHWs particularly in relation to hospitalization [9]. Our findings that Hispanics hospitalized with IBD are significantly younger than their NHW counterparts is notable, as there is limited prior data available for comparison. Prior outpatient studies have shown the opposite trend with the onset of IBD in Hispanics being significantly later than their NHW counterparts [10, 11]. However, this difference may be in fact related to a delay in diagnosis among other potential causes as foreign-born Hispanics have been found to have an older age of diagnosis than US-born Hispanics [12]. A possible explanation of our unique findings could be that Hispanic patients have more acute, as opposed to smoldering, presentations of their IBD requiring earlier presentation and hospitalization. We also consider socioeconomic factors to be at the root of this finding as Hispanics are less likely to access specialty outpatient care given a higher likelihood of being uninsured and thus more likely to present acutely to the hospital [13].

In terms of hospital course, we found that there was no significant difference in median length of stay or ICU admission between the two cohorts; however, interestingly, we noted significantly higher mortality among NHWs with IBD. The higher rates of mortality could correspond to the higher rates of surgeries among NHWs with IBD (notably small bowel surgery among those with CD and total colectomy among those with UC) or the significantly higher steroid use appreciated among NHWs, both of which have well-documented associations with higher rates of mortality [14, 15]. Older age seen among NHWs certainly could also play a factor in mortality [16]. While requiring more research, the findings of increased steroid use and surgeries may suggest that NHWs with IBD who require hospitalization have more recalcitrant and difficult-to-treat disease than their Hispanic counterparts.

*C. diff* infection is well characterized in IBD and is known to occur more frequently in UC patients, with an association for longer hospitalization, higher rates of colectomy, and higher rates of mortality [17–19]. Interestingly, we saw higher rates of *C. diff* infection among NHWs hospitalized with UC compared to their Hispanic counterparts, which to our knowledge has not previously been described. It is difficult to ascertain the underlying cause based on our study as this could be related to the higher steroid use among NHWs, or due to socioeconomic effect as noted in a large national study in 2015 that patients who had higher incomes or private insurances, as in the NHWs in our group, had higher rates of *C. diff* infection [20].

We also appreciated that Hispanic patients with IBD have significantly higher hospitalization charges compared to their NHWs counterpart. This was surprising considering there was no significant difference in LOS between NHWs and Hispanics, as well as NHWs being significantly older and having higher rates of surgery, with the latter being linked to up to 50% higher charges per admission [21]. However, this corresponds to similar findings to a similar NIS study looking at an earlier time period that also showed higher charges for both Hispanics and Asians with IBD compared to their NHWs counterparts [8]. Higher hospitalization charges have been well-documented in the Hispanic population in non-IBD hospitalizations as well as has the groups’ lower rates of access to outpatient care [22–26]. One possible explanation that would require further evaluation is that during hospitalizations for a primary diagnosis, such as IBD, patients are provided with what otherwise should be outpatient care resulting in increased charges.

While there can be an abundance of benefits to using the largest national inpatient database to help better characterize IBD in Hispanics, some limitations do exist. The major limitation of our study is the nature of the NIS dataset which is based around hospitalizations as opposed to patients themselves, which creates challenges in accurately capturing disease characteristics as well as any subsequent outcomes. Our use of more recent datasets from NIS expands on prior studies by being able to highlight the socioeconomic differences in this cohort that were previously not well described in hospitalized IBD patients. However, by the intrinsic nature of using a dataset based on ICD coding, errors in the input of codes could have occurred during the development of this dataset which also may affect the accuracy of the data. Despite the aforementioned limitations, we believe that our study helps to further the understanding of IBD in a population that has been consistently underrepresented in large national studies.

In conclusion, among a large inpatient United States database, we found that Hispanics hospitalized with IBD are younger, more likely to have lower incomes, more likely to be uninsured, and have lower rates of mortality, yet higher hospital charges than their NHW counterparts. Overall, IBD remains grossly understudied in minority groups in the United States. We hope that our study findings launch a paradigm shift in IBD research and our understanding of the disease in Hispanic Americans.

## Data Availability

Data is provided within the manuscript or supplementary information files.
